# Real case of primitive embryonal duodenal carcinoma in a young man

**DOI:** 10.3748/wjg.v23.i4.730

**Published:** 2017-01-28

**Authors:** Julien Barbieux, Riccardo Memeo, Vito De Blasi, Sebastian Suciu, Vanina Faucher, Gerlinde Averous, Catherine Roy, Jacques Marescaux, Didier Mutter, Patrick Pessaux

**Affiliations:** Julien Barbieux, Riccardo Memeo, Vito De Blasi, Jacques Marescaux, Didier Mutter, Patrick Pessaux, Hepato-Biliary and Pancreatic Surgical Unit, IRCAD-IHU, University of Strasbourg, Place de l’Hôpital, 67091 Strasbourg, France; Sebastian Suciu, Department of Gastroenterology, NHC, University of Strasbourg, Place de l’Hôpital, 67091 Strasbourg, France; Vanina Faucher, Catherine Roy, Department of Radiology, NHC, University of Strasbourg, Place de l’Hôpital, 67091 Strasbourg, France; Gerlinde Averous, Department of Pathology, NHC, University of Strasbourg, Place de l’Hôpital, 67091 Strasbourg, France

**Keywords:** Embryonal carcinoma, Germ cell tumour, Duodenum, Young male, Pancreaticoduodenectomy

## Abstract

We report here the case of a young man suffering from a rare germ cell tumour. The patient was a 25-year-old man who was referred to our centre for asthenia, stinging epigastric pain, and an iron deficiency anaemia. Gastroscopy revealed a circumferential vegetating lesion on the second portion of the duodenum. The lesion was indurated at the third portion of the duodenum, responsible for a tight stenosis. A computerized tomography-scan of the chest, abdomen and pelvis, and a pancreatic MRI showed a circumferential lesion with a bi-ductal dilatation (*i.e*., of the common bile duct and Wirsung’s duct) without metastatic localisation. The patient underwent a pancreaticoduodenectomy with lymph node dissection including all cellular adipose tissues of the hepatic pedicle from the hepatic common artery and of the retroportal lamina. Histological findings were suggestive of a duodenal embryonal carcinoma with pancreatic infiltration. This is the second published case highlighting the duodenal primitive localisation of an embryonal carcinoma with pancreatic infiltration.

**Core tip:** Duodenal embryonal carcinoma is a rare germ cell localisation. This lesion may be revealed by a chronic or acute haemorrhage. Our patient presented with an iron deficiency anaemia associated with asthenia and epigastric pain. Imaging studies and endoscopy showed a tight stenosis of the third portion of the duodenum with a circumferential lesion responsible for a common bile duct and Wirsung’s duct dilatation without any metastatic localisation. The patient underwent a pancreaticoduodenectomy and histological findings helped to identify a duodenal embryonal carcinoma with pancreatic infiltration.

## INTRODUCTION

In young men, aged between 15 and 35, testicular cancer is the leading cause of neoplasia, with an incidence rate of 2.1 for 100000[1]. It should be noted that about 5% of these patients may present with a metastatic localisation on the digestive tract[2]. The most frequent origin for embryonal carcinoma is testicular (33% of cases), as confirmed by the literature[1]. The pineal gland, the mediastinal region, the digestive tract, the lungs and the retroperitoneum could well be the primitive origin of an embryonal carcinoma[3].

Here, we reported the case of an embryonal duodenal carcinoma with pancreatic infiltration.

## CASE REPORT

A 25-year-old Bulgarian man was referred to our centre by his regular medical doctor for a strong asthenia, which lasted for the past 3 wk, and stinging epigastric pain, which was paroxysmal with dorsal irradiation responsible for nocturnal awakening getting worse since 1 mo.

The patient had neither lost weight recently nor did he present with anorexia.

Apart from a moderate active smoking, he did not have any significant surgical and medical history.

Clinical examination showed that the patient has a body mass index of 19. Examination of the abdomen revealed an epigastric sensitivity without any abdominal mass. Bowel movements were regular; however, dark stools were noted for the last week. A digital rectal exam revealed neither mass nor blood. The patient was afebrile and presented with a marked skin pallor.

Biologically, the patient presented with haemoglobin at 6.8 g/dL in relation with an iron deficiency anaemia (serum iron: 2 μmol/L, mean corpuscular volume: 76.5 fL and ferritin: 4 μmol/L). For this reason he was transfused 4 units of packed red blood cells as soon as he was admitted to our department. There was no inflammatory syndrome (leukocytes: 8.88 Giga/L and C-reactive protein: 22.3 mg/L). Liver function test results showed a cytolysis (glutamic pyruvic transaminase: 218 U/L and glutamic oxaloacetic transaminase: 76 U/L) as well as an anicteric cholestasis (gamma-glutamyl transferase: 644 U/L, alkaline phosphatase: 507 U/L and total bilirubin: 2.7 μmol/L). Lipase was at 2591 U/L and quickly decreased. Tumoural markers were measured: carcinoembryonic antigen: < 1 μg/L; carbohydrate antigen 19-9: 17.2 kU/L; alpha foetoprotein: 2.1 μg/L; β human chorionic gonadotropin < 3 UI/L; lactate dehydrogenase: 117 U/L.

**Figure 1:**
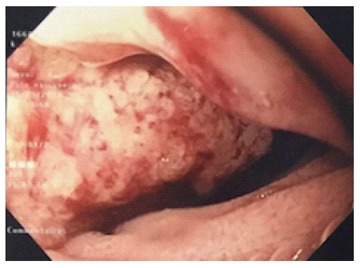
Gastroscopy shows a circumferential vegetating mass with a villous appearance on the second portion of the duodenum.

Rectoscopy performed until 40 cm from the anal margin did not show anything specific. Gastroscopy has revealed a circumferential vegetating lesion with a villous appearance on the second portion of the duodenum (Figure 1). This lesion became indurated and ulcerated at the third portion of the duodenum and was responsible for a tight stenosis. Biopsy findings were evocative of a slightly differentiated adenocarcinoma of biliopancreatic origin (cytokeratin 7+ and cytokeratin 20-).

**Figure 2:**
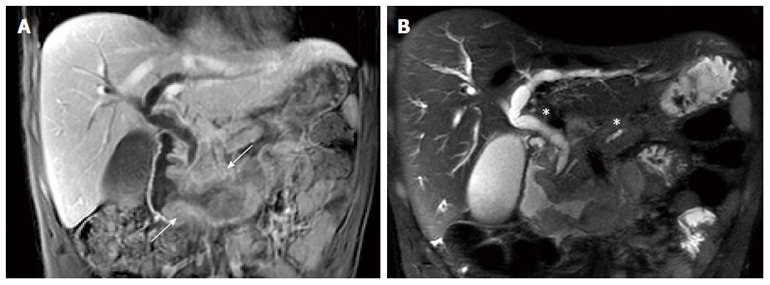
Magnetic resonance imaging. A: MRI, coronal T1-weighted MR image with contrast showing the duodenal tissue thickening spreading from the second duodenum proximal part up to the duodenojejunal flexure (white arrow); B: MRI, coronal T2-weighted MR image showing the pancreatic duct and bile duct upstream swelling (white asterisks) without any secondary hepatic lesion. MRI: Magnetic resonance imaging.

A computerized tomography (CT) of the chest, abdomen and pelvis showed a circumferential lesion thickening of up to 2 cm at the level of the second and third portions of the duodenum with a bi-ductal dilatation (of the common bile duct and main pancreatic duct (Figure 2). An 8 mm adenomegaly could be noted in a retropancreatic position. No secondary lesion was observed. Magnetic resonance imaging (MRI) of the pancreas and magnetic resonance cholangiopancreatography (MRCP) confirmed this duodenal tissue thickening spreading from the proximal part of the second portion of the duodenum up to the duodenojejunal flexure, which was accountable for pancreatic duct and bile duct upstream swelling without any secondary hepatic lesion (Figure 2). Some very short contact adenomegalies were also observed there.

**Figure 3:**
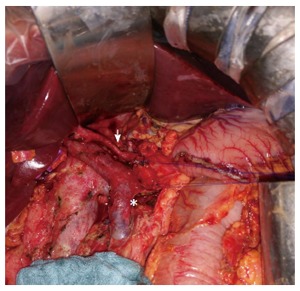
Intraoperative view of the pancreaticoduodenectomy showing lymph node dissection with excision of all cellular adipose tissues of the hepatic pedicle from the common hepatic artery (white arrow) and of the retroportal lamina (white asterisk).

**Figure 4:**
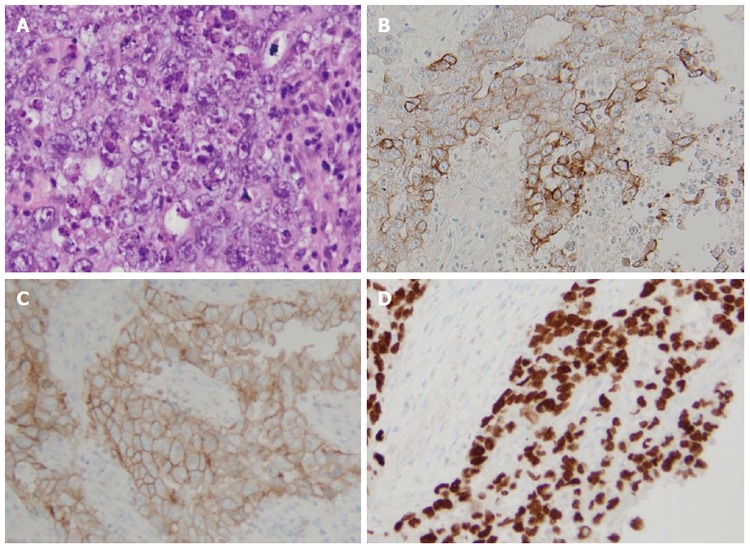
Histological findings of the operative specimen showing an embryonal carcinoma: pleomorphic cell proliferation with marked cytonuclear atypia, granular or clear cytoplasm, arranged in nests or solid pattern, associated with a fibrous stroma and an abundant lymphocytic inflammatory infiltrate (A). Specimen cells expressing cytokeratin 7 (B), CD30 (C) and SALL4 (D) are evidenced in immunohistochemical staining. A: HE × 400; B: Cytokeratin 7 × 200; C: CD30 × 200; D: SALL4 × 200.

After a multidisciplinary meeting, pancreaticoduodenectomy was decided. Surgery was performed by RM. Intraoperatively, examination of the abdominal cavity did not find any peritoneal carcinomatosis or any other tumoural lesions. Picking analysis of para-aortic lymph nodes and two frozen section examination at the level of the upper part of the mesenteric pedicle did not help to identify any sign of malignancy. A pancreaticoduodenectomy was performed with a pancreaticogastric anastomosis and a hepatico-jejunal anastomosis. Lymph node dissection included all cellular adipose tissues of the hepatic hilum from the common hepatic artery and the retroportal lamina (Figure 3). Histological findings were suggestive of a duodenal embryonal carcinoma with pancreatic infiltration rated T4N0 (0 out of 48 lymph node) M0 R0 (Figure 4A). Specimen cells expressed cytokeratin 7 (Figure 4B), CD30 (Figure 4C) and SALL4 (Figure 4D) in immunohistochemical staining.

In order not to undermine a primitive cancer, a testicular ultrasound and CT-scan of the head and neck were performed without any repeat finding of the tumoural lesion. The case was presented to the oncology staff in order to put forward an adjuvant chemotherapy after cryogenic sperm preservation.

## DISCUSSION

Melanoma, renal, mammary, bronchopulmonary, gastric or testicular neoplasia[4] represent the most frequent metastatic tumours at the level of the small bowel, and particularly at the level of the duodenum. However, in young men, a testicular origin is to be favoured due to the preferential epidemiology of these cancers in this population group[1]. It should be noted that tumours with testicular germ cells are dichotomized between seminomas and non-seminomatous germ cell tumours, such as embryonary carcinomas and teratomas[5]. The sites of dissemination of testicular embryonary carcinomas which are most frequently mentioned are the lungs, the liver and the retroperitoneal space. The digestive tract provides a rare but known dissemination area (5%)[1]. Fu et al[6] reported the case of a young patient with a symptomatic duodenal metastasis which induced low digestive bleeding. On the other hand, we did not highlight any acute haemorrhagic episode but rather an iron deficiency with chronic haemorrhagic suffusion. The literature shows that patients presenting with bowel metastasis of an embryonic cancer develop, in most cases, a haemorrhagic symptom (14 out 15 patients in the study by Fu et al[6]) or a digestive occlusion[6,7]. Tumoural dissemination by contiguity is the most frequently identified tumoural expansion mode at the expense of haematogenic or lymphatic pathways[6]. This accounts for the pancreatic lesions found in our case.

A primitive duodenal tumour is a hypothesis which must be discussed because clinical and imaging examinations have not led to the identification of another primitive lesion. A duodenal primitive embryonal carcinoma was already described in a young man[8]. The hypothesis of a retro-differentiation of adenocarcinoma cells at the level of embryonal ectodermal cells of a metaplasia or differentiation in trophoblastic precursor seems to explain the histology of this primitive embryonal duodenal tumour[9]. As well as metastasis with choriocarcinoma of testicular origin, the tumoural cellular profile is not homogeneous[1]. This accounts for the biopsy initial result matching with a limited sample of the tumoural lesion. This last one was evocative of an adenocarcinoma with a biliopancreatic profile of an embryonal carcinoma at the final review.

Regarding tumour markers, high rates of β human chorionic gonadotropin are frequently described with non-seminomatous tumours, particularly choriocarcinomas. The alpha foetoprotein seems rather ascending in case of a non-seminomatous lesion, particularly embryonal carcinomas with a yolk sac differentiation[5]. We did not notice any modification of tumoural markers in our case as opposed to the case described by Küçüköner et al[8].

In conclusion, the onset of a strong anaemia in a young man requires endoscopic and imaging explorations in order to search for an embryonal tumoural lesion on the digestive tract since they are very frequently revealed by a chronic or acute haemorrhage.

## COMMENTS

### Case characteristics

A 25-year-old Bulgarian man was referred to our centre for a strong asthenia, and stinging epigastric pain getting worse since 1 mo.

### Clinical diagnosis

Clinical examination showed that the patient has a marked skin pallor with epigastric sensitivity without any abdominal mass nor recent weight loss or anorexia.

### Differential diagnosis

A slightly differentiated adenocarcinoma of biliopancreatic origin or another germ cell tumour.

### Laboratory diagnosis

The patient presented with haemoglobin of 6.8 g/dL in relation with an iron deficiency anaemia, associated with a cytolysis as well as an anicteric cholestasis.

### Imaging diagnosis

Radiological exams showed a circumferential lesion thickening of up to 2 cm at the level of the second and third portions of the duodenum with a bi-ductal dilatation without any secondary lesion.

### Pathological diagnosis

Histological findings were suggestive of a duodenal embryonal carcinoma with pancreatic infiltration associated with, in immunohistochemical staining, a specimen cells’ expression of cytokeratin 7, CD30 and SALL4.

### Treatment

After a multidisciplinary meeting, pancreaticoduodenectomy was decided and performed.

### Related reports

This is the second published case highlighting the duodenal primitive localisation of an embryonal carcinoma with pancreatic infiltration.

### Experiences and lessons

A pancreatic tumour in a young man may not be of biliopancreatic origin and requires extensive investigations to not ignore the uncommon entities as a germ cell tumour.

### Peer-review

This is an important case report of a most unusual embryonal tumour involving the duodenum and adjacent pancreas and needs publication.

## References

[1] Vardaros M, Subhani M, Rizvon K, Gotlieb V, Mustacchia P, Freedman L, Garg V, Singh J, Siddiqui G (2013). A case of gastrointestinal bleeding due to duodenal metastasis from a testicular choriocarcinoma. J Gastrointest Cancer.

[2] Chait MM, Kurtz RC, Hajdu SI (1978). Gastrointestinal tract metastasis in patients with germ-cell tumor of the testis. Am J Dig Dis.

[3] Yokoi K, Tanaka N, Furukawa K, Ishikawa N, Seya T, Horiba K, Kanazawa Y, Yamada T, Ohaki Y, Tajiri T (2008). Male choriocarcinoma with metastasis to the jejunum: a case report and review of the literature. J Nippon Med Sch.

[4] Köksal AS, Kayaçetin E, Torun S, Güneş ZE, Zengin NI (2013). An elusive etiology of upper gastrointestinal bleeding in a young man: testis tumor. Surg Laparosc Endosc Percutan Tech.

[5] Rajpert-De Meyts E, McGlynn KA, Okamoto K, Jewett MA, Bokemeyer C (2016). Testicular germ cell tumours. Lancet.

[6] Fu S, Avezbakiyev B, Zhi W, Kodali S, Rizvon K, Alaverdian A, Freedman L, Mejia J, Shahzad G, Gotlieb V (2012). Germ cell cancer presenting as gastrointestinal bleeding and developing brain metastases: case report and review of the literature. Future Oncol.

[7] Rodriguez-Lopez M, Velasco-López R, Mambrilla-Herrero S, Bailon-Cuadrado M, Plua KT, Diez-González LM, Blanco-Álvarez JI, Asensio-Díaz E, Gonzalo-Martín M, Pérez-Saborido B (2015). Duodenal involvement by seminomatous tumors. Rev Esp Enferm Dig.

[8] Küçüköner M, Kaplan MA, İnal A, Uçmak F, Firat U, Işikdoğan A (2013). Germ cell tumor in duodenum. Turk J Gastroenterol.

[9] Noguchi T, Takeno S, Sato T, Takahashi Y, Uchida Y, Yokoyama S (2002). A patient with primary gastric choriocarcinoma who received a correct preoperative diagnosis and achieved prolonged survival. Gastric Cancer.

